# Retraction: Hong, M. et al. Acetylshikonin Sensitizes Hepatocellular Carcinoma Cells to Apoptosis through ROS-Mediated Caspase Activation. *Cells* 2019, *8*, 1466

**DOI:** 10.3390/cells9081814

**Published:** 2020-07-31

**Authors:** Cells Editorial Office

**Affiliations:** MDPI, St. Alban-Anlage 66, 4052 Basel, Switzerland; cells@mdpi.com

The Editorial Office and the authors have taken the decision to retract the published article [1]. The images in Figures 2, 6 and 8 contain identical cells with rotations. These errors were reported by two readers of the journal. The authors would like to acknowledge both readers for pointing out such important mistakes. The authors apologize for not using the final data to conduct the analysis, producing unreliable results. The academic editors of *Cells* have checked this case and approved the retraction. Both readers have been informed about the retraction decision. The published paper [1] will therefore be marked as retracted.

MDPI is a member of the Committee on Publication Ethics and takes its responsibility to publish only high-quality research seriously. We regret that this issue was not identified earlier and apologize to readers of the journal.
